# Nelson Hospital

**Published:** 1912-11-16

**Authors:** 


### The Editor's Letter-Box.

### Nelson Hospital.

WITH reference to the plan of the Nelson Hospital published in our issue of September 21, Mr. R. A. Hinds, F.R.I.B.A., requests us to state that the building was designed by Mr. Francis Hatch, Lic.R.I.B.A., and carried out by Mr. Hinds. We must adhere to our criticisms, which were mainly administrative, and have to do mostly with considerations of hygiene, comfort, and reasonable privacy.—ED. THE HOSPITAL.

